# Analysis of personal psychological motivation and social psychological motivation of Retaliatory Justice under moral public opinion: Chinese cases

**DOI:** 10.3389/fpsyg.2022.1021577

**Published:** 2022-12-09

**Authors:** Xi Liu, Xiaoying Zhao, Baomin Wang

**Affiliations:** ^1^School of Law, Xi'an Jiaotong University, Xi'an, Shaanxi, China; ^2^School of Foreign Studies, Xi'an Jiaotong University, Xi'an, Shaanxi, China

**Keywords:** Retaliatory Justice, psychological motivation, social psychology, Self-Oriented Motivation, Society-Oriented Motivation, Perception of Judicial Justice

## Abstract

From a social psychology perspective, this study explored the personal and social psychological motivation of Retaliatory Justice. This study constructed a four-dimensional model of the psychological motivation of Retaliatory Justice from the two dimensions of “Self vs. Society” and “Identity vs. Resource.” They were Identity-Based Self-Oriented Motivation (Pacifying Outrage), Resource-Based Self-Oriented Motivation (Occupying Resource), Identity-Based Society-Oriented Motivation (Value Confirmation), and Resource-Based Society-Oriented Motivation (Deterrence and Control). In this study, 497 sets of valid data were extracted from 6 universities in Xi'an, Shaanxi Province, China, using 3 sets of situational experiments and the “Questionnaire Star” online survey platform as a vehicle to explore the personal motivations and psychosocial motivations of Retaliatory Justice. The empirical results showed that the situational experiment significantly verified the existence of Pacifying Outrage and Value Confirmation, and partially verified the existence of Deterrence and Control and Occupying Resource. Multiple linear regression analysis showed that only the effect of Pacifying Outrage on Retaliatory Justice showed a main effect, and the interaction between Pacifying Outrage and Deterrence and Control was not significant. Pacifying Outrage significantly affects Retaliatory Justice, while Occupying Resource significantly affects Retaliatory Justice, but the interaction between the two was not significant. Value Confirmation significantly affects Retaliatory Justice. Value Confirmation and Deterrence and Control synergistically affect Retaliatory Justice, but Deterrence and Control had no significant effect on Retaliatory Justice. Taking emotions as clues, this paper discussed the realistic value of the rheological paths of the psychological motivation of Retaliatory Justice, which brought enlightenment to the improvement of social morality, the cultivation of judicial trust, and the construction of psychological service system.

## Introduction

Whenever there is a mass focus on the flow of Internet information, a public opinion event will be formed. The rapid development of public opinion events and the wide range of participants fully reflect the public's social perception and concern for judicial justice and construct the overall impression of judicial justice about the society. The Perception of Judicial Justice is based on the individual's outlook on life and values, influenced by the concepts of others, and fueled by social emotions, and finally produces the Perception of Judicial Justice (Géa et al., 2022; Indradevi, 2022). The Perception of Judicial Justice is not simply right and wrong, good and evil, but a subjective perception strengthens the social connection among evil deeds, evil people, evil consequences, and punishment (Trood et al., 2021). Therefore, in this sense, the Perception of Judicial Justice can be translated as Perception of Retaliatory Justice (Ng, 2020). From the semantic concept, Retaliatory Justice has two meanings, namely, “Retaliation” and “Justice.” Later, the semantic concept of Retaliatory Justice was translated into academic concepts, its core connotation was further strengthened, and various academic concepts with different focuses emerged under different disciplines. For example, its focus in ethics is on Socio-Ethical values (Cheng et al., 2021), while in jurisprudence, it focuses on the purpose and value of punishment (Peterson and Allamong, 2022; Shi et al., 2022), and so on. In general, Retaliatory Justice refers to the punishment of subjects who transgress norms, offend morality, and commit harm, and to the knowledge, the aforementioned punishment can lead to a state of “deserving” and “deserving punishment” in society (Wang et al., 2021; Zhang et al., 2022). At the microlevel, Retaliatory Justice is the knowledge of punishment; at the macrolevel, Retaliatory Justice is the knowledge of the state of society in which “good and evil are rewarded.” From a psychological point of view, Retaliatory Justice is an inherent demand for crime and punishment, emphasizing the subjective level of fair evaluation. From a sociological point of view, Retaliatory Justice is an external need for the judicial system, emphasizing the objective guarantee of justice (Ghossoub and Felthous, 2020; Student and Abdulla, 2021). Based on the above characteristics and after analyzing the semantic concept and academic connotation of Retaliatory Justice, this study focuses on social psychology (or legal psychology) to explore the psychological mechanism of Retaliatory Justice with the psychological motivation of Retaliatory Justice as the core of the study and “converge” different theoretical bases of research in order to better “diffuse” and “radiate” Retaliatory Justice research in different disciplines, categories and topics (Tan et al., 2021; Beiser-Mcgrath et al., 2022; Wei et al., 2022).

During the COVID-19 epidemic, a “fake vaccine incident” with a wide range of influence, high social concern, and strong public sentiment occurred. However, the public's request to severely punish the companies involved and the dereliction of duty did not receive a positive response, nor did the trial result publicize the public outrage (Serres et al., 2022). Every judicial case of high social concern is an opportunity for the improvement of the social governance system and self-examination, and Retaliatory Justice just starts from the needs of the people and points out the direction for institutional reform and social change (Liu, 2021). Throughout the occurrence and evolution of public opinion events related to judicial fairness, extensive participation of the public often only attracts widespread attention and does not really lead to extensive or profound social changes. Since the social effects of public opinion events are constantly diluted by massive amounts of information, there are very few cases that can truly form a transformative social effect (Schwabe et al., 2020; Yuan and Chen, 2021). Why is it that when a judicial case occurs, it can only attract attention and heated discussions from all walks of life in a short period of time, but after the emotional catharsis, everything returns to its original state. Obviously, the psychological motivation of Retaliatory Justice lacks the “stamina” to affect social change (Li and Yang, 2020). Therefore, it is necessary to gain an in-depth understanding of the inner motivation of Retaliatory Justice.

For the value of academic research, Retaliatory Justice is affected not only by the individual level, such as moral awareness, emotional experience, and perception of justice, but also by the social level, such as institutional arrangements, judicial practices, and political situations. Therefore, Retaliatory Justice combines various social knowledge categories and psychological experiences and is a multidisciplinary and interdisciplinary Research Topic. From the perspective of philosophy, Retaliatory Justice is related to the current political system and judicial system and is closely related to political philosophy, moral philosophy, and legal philosophy (Deutsch, 1975; Tyler, 1989). From the perspective of psychology, Retaliatory Justice is the personal feeling about the breakthrough of moral indignation, which is closely related to moral psychology and emotional psychology. From the scope of sociology, the feeling of Retaliatory Justice originates from judicial cases and social events and is closely related to social control and social justice (Folger and Bies, 1989). Therefore, research on Retaliatory Justice requires researchers to conduct interdisciplinary integration and multidisciplinary interpretation, which can provide references for interdisciplinary social science research.

For the value of social practice, the study of Retaliatory Justice can provide important inspiration for rationally guiding social emotions and reasonably relieve irrational collective emotions (Carlsmith et al., 2002). At the same time, Retaliatory Justice is essentially a social psychological phenomenon combined with social life practice, and its appeal is based on the reflection and dissatisfaction with the current social system. The study of Retaliatory Justice can explore the negative life experience and social experience of the people and answer the reflected questions of social psychological service and appeal, so as to build a healthy social psychological service practice system (Balvig and Gunnlaugsson, 2015).

### Innovation points

1. Domestic and international research on Perception of Judicial Justice lacks systematicity, and the introduction of Retaliatory Justice helps to improve the conceptual system and research framework of Perception of Judicial Justice. The lack of research reference literature for conducting localized Retaliatory Justice research in countries around the world is both a difficulty and an innovation in this study. To a certain extent, this study brings a new concept, form, and issue of justice into the researcher's perspective, focusing on the psychological mechanisms and social effects behind it and linking the micro- and macro-academic perspectives.

2. The interdisciplinary nature of the issue of Retaliatory Justice is evident in the context of social psychology. There is a need to link multiple disciplines in terms of theory and research variables to make interdisciplinary research possible. In a multidisciplinary and interdisciplinary research, Retaliatory Justice can co-construct social systems that are long-lasting and applicable to social development, such as judicial systems, journalism systems, psychosocial service systems, and social governance systems.

3. A healthier and benign social mechanism is promoted. Exploring the social psychological motives of Retaliatory Justice helps to understand the outbreak and evolution patterns of social morality and emotion in public opinion events, as well as the social psychological mechanisms and motivating factors of Retaliatory Justice in public opinion events, integrating social psychology, social emotion, social morality, and social governance. With the mastery of the social psychological motives of Retaliatory Justice, it helps to provide theoretical paths for guiding, managing, intervening, and relieving negative public opinion and bad social mentality, and provides practical possibilities for building, improving, and innovating social governance and public opinion management mechanisms.

The Section Introduction is the research background and research significance. The Section Identity-based and resource-based psychological motivation and research hypotheses explains the core connotation of Retaliatory Justice and constructs a four-dimensional model of the psychological motivation of Retaliatory Justice from the two dimensions of “Self vs. Society” and “Identity vs. Resource.” The Section Methods and experimental studies sets up three groups of situational experiments to empirically test the quaternary model of the psychological motivation of vindictive justice. The Section Discussion of empirical results discusses the analytical results of the empirical test. The Section Rheological path of the psychological motivation of Retaliatory Justice couples the development of events, emotional changes and the psychological motivation of Retaliatory Justice and sorts out the rheological path of the psychological motivation of Retaliatory Justice. The Section Conclusion is the conclusion.

## Identity-based and resource-based psychological motivation and research hypotheses

### Pacifying Outrage: Identity-based Self-Oriented Motivation

“Pacifying Outrage” is the content represented by the original moralist view; that is, Retaliatory Justice is induced and promoted by moral emotions, of which moral outrage is the main component. Pacifying Outrage mainly focuses on the self-emotional experience of victims and bystanders to the injury event, which is a unilateral, independent, and situation-specific psychological response (Rone, 2021). When individuals experience injustices, the internalized moral norms for individuals to participate in social life are broken, thereby activating strong moral emotions. Retaliatory Justice can alleviate the moral indignation of individuals and can also alleviate the cognitive dissonance caused by moral concepts (Gonick and Sophie, 2016). In general, the Retaliatory Justice motivated by the pacification of Pacifying Outrage represents the efforts made by oneself to maintain moral values, and its ultimate effect is to avoid the influence of negative moral emotions and alleviate the state of moral cognitive dissonance (Ju and You, 2020).

Righteous indignation is the emotional core of Pacifying Outrage as the psychological motivation of Retaliatory Justice. This means that righteous indignation is the main emotional experience of Retaliatory Justice in cognition, and there are both Cognitive Path 1: “Fair Cognition → Moral Cognition → Righteous Indignation,” but also Cognitive Path 2: “Righteous Indignation → Moral Cognition → Fair Cognition.” In Cognitive Path 1, when members of society experience injustice (Purnell, 2019), a strong sense of injustice is aroused. Individuals develop resentment toward the moral perception of “just imbalance” and continue to amplify their perception of immorality, which eventually develops into anger, that is, Outrage. Moral outrage is indeed an important factor in measuring and influencing perceptions of justice, and it positively affects Retaliatory Justice (Koak, 2021; Lin and Loi, 2021). Therefore, an injustice will activate the outrage of the object (victims and bystanders) of the harmful behavior against the subject (perpetrators). In Cognitive Path 2, Pacifying Outrage will react on moral perception and moral judgment and even amplify the results of moral judgment. When Pacifying Outrage is on the rise, almost all attention is focused on the moral perception of harmful behavior (Antadze, 2020; Hart et al., 2020). Obviously, intentional harmful behavior violates social moral values, breaks the social balance that relies on moral norms to restrain one's own behavior, and also destroys the identification and adherence of other group members to moral norms (Sawaoka and Monin, 2020). Therefore, what degree of punishment can be called fair, and the answer to this question needs to be based on the judgment and measurement of the degree of moral imbalance, that is, the state of Retaliatory Justice. Therefore, it can be assumed that the higher the pacification of Pacifying Outrage activated by a crime, the stronger the Retaliatory Justice demands activated by the crime, namely:

H1: The higher the pacification of Pacifying Outrage activated by a crime, the more severe the punishment for that crime.

### Occupying Resource: Resource-based Self-Oriented Motivation

For Retaliatory Justice, whether it is based on emotional outrage or rational deterrence, both starting points have been widely recognized. But are there other psychosocial motivations for understanding Retaliatory Justice? Resource-Based Self-Oriented Motivation is a widely overlooked perspective (Yang et al., 2007). “Occupying Resource” refers to the relationship between the presence and absence of harmful behaviors that determines the moral status of individuals. Those who do not harm are always in a higher moral position than those who do harm, and they have a sense of moral superiority. According to Goffman's Stigma Theory, the ultimate purpose of stigma is to marginalize other groups in the process of resource allocation in order to maximize their own interests (Poteat et al., 2013). Marginalization through moral superiority not only expresses stronger interest demands in the process of resource redistribution, but also eliminates the negative emotions generated in the process of stigmatizing and marginalizing the morally inferior (Li et al., 2022).

The emotional core of Occupying Resource as the motivation of Retaliatory Justice is superiority; to be precise, it should be moral superiority. The reason why it is named morality is that the harmful behavior is a behavior that results in harming the moral order (Safi et al., 2022). When a case occurs, bystanders will spontaneously establish a moral level sequence in their hearts, forming a condescending posture, “looking down” on the moral status of the perpetrator. Law and morality are constantly approaching and will eventually become a legal system with inherent moral values. Although morality and law are two different norms, there are areas that cannot be governed by each other, but law is moral (Jolex and Kaluwa, 2022). Therefore, criminals violate the moral beliefs generally recognized by the society and “consume” others' trust and recognition of their own morality. Furthermore, for those who have not violated the law, their moral level naturally remains in a state of “unconsumed.” Therefore, the first source of moral superiority lies in the fact that the harmful behavior occurred (Mor, 2022; Yang et al., 2022).

In addition to the above reasons, cognitive bias is also one of the sources of moral superiority. In the face of the fact of illegal and criminal behavior, a harmful behavior is likely to be interpreted as the conduct of the perpetrator, that is, to make a negative moral judgment on the perpetrator (Doolan and Bryant, 2021). For most people, when they hear the facts of a crime, they have less chance and possibility to understand the whole process of the incident in detail, so it is easy to fall into the moral judgment of the perpetrator, believing that their moral level is low (Ren et al., 2021). The result is an inappropriate construction of the moral status of both parties under biased perceptions. Interestingly, when performing Self-Evaluation, individuals generally believe that their moral level will be higher than the average level of the society as a whole (Nieto and Vazquez, 2021; Schubert et al., 2021). This phenomenon is called Overly Positive Self-Evaluation or Better-Than-Average Effect. This cognitive tendency further adds to the disparity in the moral status of bystanders and perpetrators. Therefore, it can be assumed that the higher the Occupying Resource activated by a crime, the stronger the Retaliatory Justice demands activated by the crime, namely:

H2: The higher the Occupying Resource activated by a crime, the more severe the punishment for that crime.

### Value Confirmation: Identity-based Society-Oriented Motivation

Identity-Based Society-Oriented Motivation is named “Value Confirmation,” which means that Retaliatory Justice sends a message to the whole society: what kind of behavior is wrong and why it is wrong. Value Confirmation is different from Deterrence and Control, and its purpose is to achieve organic integration and close unity of society and ultimately create a harmonious and healthy social development state (Leonard, 2022; Willis and Hoyle, 2022). This is a positive, constructive, and future-oriented psychological motivation. The foundation of social rule of law construction lies in the people's recognition of the rule of law and the recognition of the social concepts and social values represented by the legal system. Retaliatory Justice is the reaffirmation and re-authentication of the social concept and social value pursued by the vast majority of people (Jeffries et al., 2021; Decker et al., 2022). Through punishment, criminals can be deeply aware of the symbolic meaning of their breakthroughs in laws and regulations, that is, the abandonment of social values, and also profoundly reshape their identification and compliance with social values. On this level, the purpose of punishment is not to deter, but to reaffirm value. Theory of Restorative Justice is a theory of active practice, emphasizing that the law and punishment systematically re-educate and reshape the behavior of evildoers, and it is a positive and active transformation paradigm (Mills et al., 2019; Tapp et al., 2020; Gavrielides, 2022). The judicial practice of Restorative Justice is often aimed at the victims and the perpetrators, and the means and links to achieve justice have nothing to do with bystanders.

In the context of Restorative Justice, when criminal behavior occurs in the collective, members of the collective will face Moral Loss: an emotional experience of sadness and disappointment. For offenders, shame is the key to realizing their self-restoration. But often, innocent people do not see themselves as the same camp as criminals, and the labels of criminals distinguish them from each other through intuitive cognitive pathways. The practice process of Restorative Justice clearly demonstrates the role and position that social values play in maintaining social fairness and justice (Lin et al., 2012). Therefore, social value is the core of Value Confirmation, and the emotional experience it activates is also generated around social value. The sense of value for social value means that social value itself has value, and it is the basis for people to feel and understand its value. For example, helping others is only a concept in the social value system, but because helping others can gain positive self-affirmation from helping others. At the same time, the beneficiaries can experience the kindness of others from the beneficiary behavior, which increases the “kindness” in the society and brings positive emotions to both parties. Therefore, the sense of value for social value is its own. An important factor in determining Value Confirmation is the perception of the level of norm recognition and the effectiveness of communication induced by the handling of a crime. Therefore, it can be assumed that the higher the Value Confirmation activated by a crime, the weaker the Retaliatory Justice appeal activated by the crime, namely:

H3: The higher the Value Confirmation activated by a crime, the less severe the punishment for that crime.

### Deterrence and Control: Resource-based Society-Oriented Motivation

The content represented by “Deterrence and Control” is the content of the original functionalist view; that is, Restorative Justice is determined by the control and deterrence function of punishment. It is generally agreed that harsh punishments can deter potential offenders and make offenders fearful of repeating offenses. These cognitions and concepts are important motivations for people to expect Restorative Justice (Kane, 2006). Deterrence and Control appeals to Restorative Justice's individual not to care about the object of punishment, but is a passive strategy to restrain all members of society. Behind the private relief, the overall social atmosphere is bound to strengthen, solidify, and deteriorate, immersed in distrust of the construction of the rule of law and doubts about social constraints (Williams, 1985). Deterrence and Control is also an impartial maintenance habit from a past perspective. It is only when evil deeds occur that the need for redemption is thought of, in stark contrast to future-oriented Value Confirmation.

The emotional core of Deterrence and Control is anxiety. Everyone hopes that their profits will not be lost and their rights will not be violated, but everyone has to face the risk of being a potential victim. Anxiety, as an emotion related to worry and pain, not only affects the individual's choice of action, but also affects the individual's spiritual world (Lutsenko, 2018; Choi, 2019). In order to relieve anxiety, individuals need and must find a tool to support their “sense of security” that they will not be violated, and an authoritative, coercive, and legitimate legal system is the preferred tool. Under the normal and effective operation of the legal system, the deterrent function of the law can effectively exert its effect and reduce the occurrence of harmful behaviors (Feld, 2006). In an absolutely ideal state, the deterrent effect of the law should be able to prevent harm from happening.

Anxiety in anxiety disorders is an abnormal psychology that affects the daily life of an individual in severe cases and refers to an individual's emotional experience (Cohen, 1984). However, the anxiety induced by the unsatisfied state of Deterrence and Control is social and has the characteristics of unchangeable, unpreventable, and untreated. The immutable characteristic means that the object of anxiety is potential, broad, and unspecified. Individuals can only narrow the scope of potential harm and cannot change the probability of harm in social reality. Therefore, the risk of being harmed always exists, it can even be considered as a certain inevitability, and the anxiety it causes has an unalterable character (Freeman et al., 2020; Kaviani et al., 2020). Second, the irreversibility is reflected in the fact that risks are inherently non-specific, and the objects of anxiety are inherently inexhaustible (Anderson et al., 1977). For example, parents worry about their children's safety on the way to and from school, but cannot exhaust all the risks they face on the road. So, anxiety caused by the risk of being exposed to a potential aggression is not preventable. Therefore, it can be assumed that the higher the Deterrence and Control activated by a crime, the stronger the Retaliatory Justice demands activated by the crime, namely:

H4: The higher the Deterrence and Control activated by a crime, the more severe the punishment for that crime.

### Four-Dimensional Psychological Motivation Model of Retaliatory Justice

**Figure 1:**
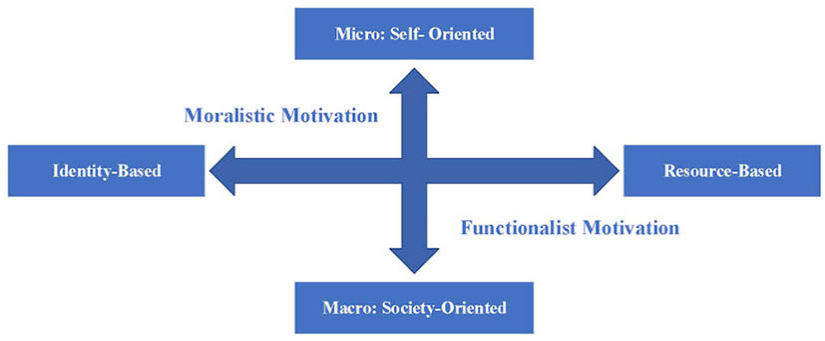
A Two-dimensional Motivation Model of Retaliatory Justice.

Moralism and functionalism focus on the micro-self and macro-society, respectively. The former takes the individual's moral outrage toward the harming behavior as the driving force and takes the individual's moral concept and value system toward the harming behavior as the object of harm. The latter takes society's potential concerns about harmful behavior as the driving force and takes the collective interests and overall harmony of society as the object of harm. Therefore, the psychological motivation model of Retaliatory Justice has the dimension of “Self vs. Society.” At the same time, the psychological motivation of Retaliatory Justice may also point to different objects; that is, there is the dimension of “Identity vs. Resource.” Therefore, the two dimensions of “Self vs. Society” (vision) and “Identity vs. Resource” (basis) can be used to locate the psychological motivation of Retaliatory Justice. Moralism and functionalism are Identity-Based Self-Oriented Motivation and Resource-Based Society-Oriented Motivation, respectively, as shown in Figure 1.

**Figure 2:**
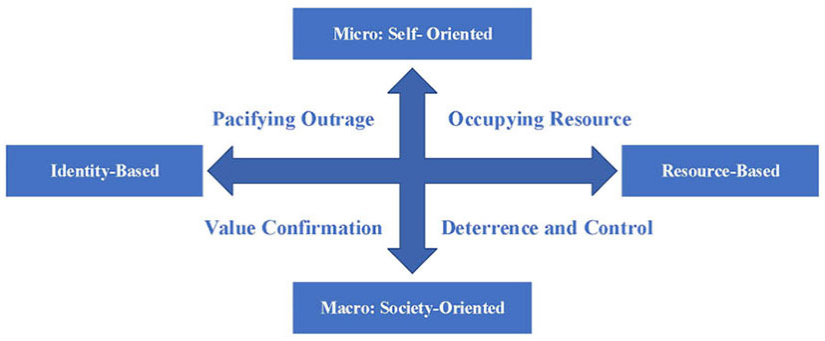
A Four-dimensional Motivation Model of Retaliatory Justice.

However, according to the Theory of Comparative Emotions, in the process of social cognition and social comparison of injustices, retaliation can alleviate negative emotions and feelings of victimization caused by injustices (Eva et al., 2017). It can be considered that calming emotions, such as calming moral outrage, fulfill a certain function. Because, calming moral outrage can alleviate the individual's cognitive dissonance and can also relieve negative and extreme social emotions, while maintaining the harmony between the individual's psychology and social atmosphere. It should be pointed out that the two-dimensional psychological motivation of Retaliatory Justice is functional. These functions can be directed to either self or society, to identity and emotion, or to resource and functions. To this end, this study uses “Pacifying Outrage,” “Occupying Resource,” “Value Confirmation,” and “Deterrence and Control” as Identity-Based Self-Oriented Motivation, Resource-Based Self-Oriented Motivation, Identity-Based Society-Oriented Motivation, and Resource-Based Society-Oriented Motivation, respectively, as shown in Figure 2.

The psychological motivation of Retaliatory Justice is classified into four dimensions according to the two dimensions of “Self vs. Society” (vision) and “Identity vs. Resource” (basis). Identity-Based Self-Oriented Motivation means that the moral standards and value norms within the collective are generally recognized and abided by. When harmful behavior breaks the norm, it will cause moral cognitive dissonance at the individual level. In order to calm moral outrage and other emotions, individuals will actively choose to impose just punishments. Resource-Based Self-Oriented Motivation means that when conflict occurs, individuals protect their own interests by imposing just punishments in order to consolidate their own power and status. Identity-Based Society-Oriented Motivation means that when the harm occurs, the cultural concepts, moral beliefs, and value connotations shared by the members of the group are violated. Therefore, it is hoped that fair punishment will be used to reshape the identity of all members of the society on moral values and norms. Resource-Based Society-Oriented Motivation refers to the widespread application of reciprocal and equal interaction rules in social interactions. Harmful behaviors break the rules of communication and threaten social order and public interests. Each individual hopes to restrain potential harmful behaviors through fair punishment.

## Methods and experimental studies

### Experimental design and situation Settings

#### Study 1

This case originated from the Qufu City Procuratorate, Shandong Province, October 2020 (http://news.jcrb.com/jsxw/2020/202010/t20201020_2214799.html) was used to test hypothesis H1 (Pacifying Outrage) and hypothesis H4 (Deterrence and Control). The following is the design of the different situation settings and identifies the questionnaire shown in Table 1.

**Table 1:** Items of Pacifying Outrage, Deterrence and Control, and Retaliatory Justice.

**Name**	**Items**	**Description**
**Dependent Variable**		
Retaliatory Justice	What do you think the severity of the punishment should be?	10-point forward scoring. The higher the score, the more severe the punishment.
**Independent Variable**		
Damage Level	How bad do you think the behavior is?	7-point forward scoring. The higher the score, the worse the behavior.
Subjective Intent	To what extent do you think ZHANG can be morally forgiven?	7-point reverse scoring. The higher the score, the more forgivable the behavior.
Reconnaissance Difficulty	What do you think is the possibility of a behavior similar to ZHANG being discovered?	7-point reverse scoring. The higher the score, the more likely the behavior will be detected.
Publicity Breadth	To what extent do you think ZHANG's case was made public?	7-point forward scoring. The higher the score, the more public the case and the wider the impact.

“*Employees use loopholes to defraud company funds*”:

*ZHANG is an employee of the financial department of a company, responsible for the company's internal fund allocation and external money transfer. ZHANG has a very good understanding of the company's financial system. ZHANG took advantage of system loopholes in operations such as money transfers to defraud the company's cash*.

High Pacifying Outrage situation:

*ZHANG often withdraws cash from the company, and the huge amount has affected the salary payment of grassroots employees. After investigation, ZHANG has been in and out of high-end clubs for a long time, living an excessively extravagant life, and participating in gambling. Huge expenses made ZHANG unable to make ends meet, and finally, through loopholes in the company's system, he extracted cash to make up for his shortfall*.

Low Pacifying Outrage situation:

*The amount of funds that ZHANG obtained from the company was relatively small, and he could fill the company's deficit by selling assets. After investigation, ZHANG was in order to provide assistance and compensation to grassroots employees who suffered work-related injuries and did not receive full compensation from the company, and finally obtained cash through loopholes in the company's system*.

High Deterrence and Control situation:

*ZHANG clearly knows that it is extremely difficult to detect the behavior of cheating the company's cash. However, in an accidental situation, ZHANG's behavior of extracting company cash was discovered by a colleague. After the colleague reported it, ZHANG was transferred to the judicial authority. A large number of media have carried out in-depth reports on ZHANG's case of cheating the company's cash, and the detailed reports have attracted a large number of readers to continue to pay attention to the progress of the case*.

Low Deterrence and Control situation:

*The company's financial system stipulates that the financial accounts must be reviewed every quarter, so ZHANG's behavior was eventually reported by the company and transferred to the judicial authority. ZHANG's case of cheating the company's cash has not attracted media attention, nor has it been reported in depth. It is difficult for readers to know ZHANG's behavior and its negative impact on the company*.

#### Study 2

This case originated from the Jiangyin City People's Court, Jiangsu Province, August 2019 (https://www.sohu.com/a/344135591_654410) was used to test hypothesis H1 (Pacifying Outrage) and hypothesis H2 (Occupying Resource). The following is the design of the different situation settings and identifies the questionnaire shown in Table 2.

**Table 2:** Items for Pacifying Outrage, Occupying Resource, and Retaliatory Justice.

**Name**	**Items**	**Description**
**Dependent Variable**		
Retaliatory Justice	What do you think the severity of the punishment should be?	10-point forward scoring. The higher the score, the more severe the punishment.
**Independent Variable**		
Damage Level	How bad do you think TIAN's behavior is?	7-point forward scoring. The higher the score, the worse the behavior.
Subjective Intent	To what extent do you think TIAN can be morally forgiven?	7-point reverse scoring. The higher the score, the more forgivable the behavior.
Moral Disparity	What do you think your moral level is? What do you think TIAN's moral level is?	7-point forward scoring. The higher the score, the higher the moral level. The difference indicates that the gap between the moral disparity of the two is greater.
Resource Allocation Constraints	To what extent do you think TIAN is restricted to his rights (like others)?	7 points forward scoring. The higher the score, the more public the case and the wider the impact.

“*Boss of a heavy metal smelting factory illegally discharges poisonous sewage*”:

*TIAN is the owner of a heavy metal smelting factory. TIAN freely discharges industrial sewage containing lead, chromium, and other heavy metals produced by the factory, which seriously pollutes the environment and affects the growth of vegetation. If people live in a polluted environment or eat contaminated food for a long time, it will cause chronic heavy metal poisoning and cause permanent organ damage*.

High Pacifying Outrage situation:

*TIAN knew that the surrounding area of the factory was a grain producing area and industrial sewage would have a serious impact on the health and safety of residents, but in order to increase the economic benefits of the factory, TIAN privately discharged it directly into the local canals used for agricultural irrigation. TIAN's behavior has been reported and will be tried by law*.

Low Pacifying Outrage situation:

*A factory in TIAN is located in an industrial area far away from the city. Since there are almost no ordinary residents living around, it is difficult for industrial sewage to directly affect people's health. TIAN saw that all the surrounding enterprises discharge industrial sewage directly without any treatment, so TIAN also directly discharged industrial sewage into the canal. TIAN's behavior has been reported and will be tried by law*.

High Occupying Resource situation:

*The crime committed by TIAN is a manifestation of moral decline, and he should be labeled as a “criminal.” In the future, he will be restricted to a certain extent in social life such as enjoying social welfare policies, and his life will be stained*.

Low Occupying Resource situation:

*Although TIAN has committed a crime, it cannot be proved that he is immoral, because everyone may have some unethical behaviors. The society should not label TIAN as a “criminal,” nor should it restrict him from enjoying his due rights in social life, such as enjoying social welfare policies*.

#### Study 3

This case originated from the Court in Guzhen, Anhui Province, November 2020 (https://new.qq.com/rain/a/20210726A08KBQ00) was used to test hypothesis H3 (Value Confirmation) and hypothesis H4 (Deterrence and Control). The following is the design of the different situation settings and identifies the questionnaire shown in Table 3.

**Table 3:** Items for Deterrence and Control, Value Confirmation, and Retaliatory Justice.

**Name**	**Items**	**Description**
**Dependent Variable**		
Retaliatory Justice	What do you think the severity of the punishment should be?	10-point forward scoring. The higher the score, the more severe the punishment.
**Independent Variable**		
Reconnaissance Difficulty	What do you think is the possibility of ZHAO's behavior being discovered?	7-point reverse scoring. The higher the score, the more likely the behavior will be detected.
Publicity Breadth	To what extent do you think the case involved in ZHAO's behavior was made public?	7-point forward scoring. The higher the score, the more public the case and the wider the impact.
Normative Recognition Level	To what extent do you think ZHAO's remarks during his detention meet the expectations of social value norms?	7-point reverse scoring. The higher the score, the higher the level of norm recognition.
Communication Effectiveness	How sincere do you think ZHAO respects the injured and apologizes in person?	7-point reverse scoring. The higher the score, the better the communication.

Situational material—”*Drink driver hits passerby*”:

*ZHAO is a sales clerk and often drinks alcohol at parties with business partners. After drinking one day, ZHAO thought that he was not drinking much and drove home by himself. A pedestrian crossing the road was struck and seriously injured while driving through the intersection. ZHAO was then arrested by the police for driving under the influence of alcohol*.

High Deterrence and Control situation:

*Recently, the government is carrying out “Drink Driving” rectification action. The mainstream media has carried out extensive publicity and coverage of drinking and driving behaviors and cases. However, the city where ZHAO lived only set up alcohol detection points at the intersections of main streets, and ZHAO was familiar with local roads and could easily avoid the detection points. If ZHAO hadn't hit the pedestrian, his drink-driving behavior would not have been discovered*.

Low Deterrence and Control situation:

*Recently, the government is carrying out “Drink Driving” rectification action. The police set up detection points for drivers drunk driving at each intersection, and it was impossible for ZHAO to escape the inspection. However, due to the good security in ZHAO's city, the media has hardly paid attention to cases of drunk driving, and the public has rarely paid attention to such reports*.

High Value Confirmation situation:

*During his detention, ZHAO contacted the injured and their families through the police, expressed deep apology to the injured and was willing to take the corresponding legal responsibility. ZHAO admitted to the victim and the police that his drinking and driving was a wrongful behavior of disregarding the lives and safety of others and asked everyone to supervise him not to do it again in the future*.

Low Value Confirmation situation:

*During his detention, ZHAO did not apologize to the injured and their families in any form and expressed his disdain for the victim by not responding or communicating. At the same time, ZHAO repeatedly said to others that “the pedestrian does not take the initiative to avoid the car, the death and injury are deserved, and the driver is the victim.”*

### Experimental objects

At present, the majority of university students in China are adults over the age of 18. According to theory of Moral Development, 18-year-old adults have completed the whole process of moral development and have formed stable moral concepts and moral cognition. University students were recruited to participate in the experimental survey at 6 universities (AA, BB, CC, DD, EE, FF University) in Xi'an, Shaanxi Province, China. The experimental investigation uses the “Questionnaire Star” network investigation platform as the carrier, and university students can participate in the investigation by scanning the QR code.

A total of 139 university students from AA University and BB University participated in and completed the experimental study 1, and finally, 133 valid samples were obtained. Among them, there are 79 women (59.4%) and 54 men (40.6%); the average age is 20.15 ± 1.21 years old, the average time is 237.36 ± 194.69 s; there were 68 urban householders (51.1%) and 65 rural householders (48.9%). A 2 (High Pacifying Outrage vs. Low Pacifying Outrage) × 2 (High Deterrence and Control vs. Low Deterrence and Control) completely randomized block design was used to test the effects of Pacifying Outrage and Deterrence and Control on Retaliatory Justice. According to Pacifying Outrage, the Damage Level and the Subjective Intent were used to evaluate the Pacifying Outrage; according to Deterrence and Control, the Reconnaissance Difficulty and the Publicity Breadth were used to evaluate Deterrence and Control. The variables are shown in Table 4.

**Table 4:** Experimental Variables of Pacifying Outrage and Deterrence and Control.

	**High pacifying outrage**	**Low pacifying outrage**	**Total (*N*)**
High Deterrent and Control	*N* = 40	*N* = 32	72
	High Damage Level, High Subjective Intent High Reconnaissance Difficulty, High Publicity Breadth	Low Damage Level, Low Subjective Intent High Reconnaissance Difficulty, High Publicity Breadth	
Low Deterrent and Control	*N* = 40	*N* = 21	61
	High Damage Level, High Subjective Intent High Reconnaissance Difficulty, High Publicity Breadth	Low Damage Level, Low Subjective Intent Low Reconnaissance Difficulty, Low Publicity Breadth	
Total (*N*)	80	53	133

A total of 227 university students from CC University and DD University participated in and completed the experimental study 2, and finally, 212 samples were obtained. Among them, 122 were women (57.5%) and 90 were men (42.5%); the average age was 20.07 ± 1.50 years old, and the average time was 199.35 ± 87.74 s; there were 97 urban householder (45.8%) and 115 rural householder (54.2%). A 2 (High Pacifying Outrage vs. Low Pacifying Outrage) × 2 (High Occupying Resource vs. Low Occupying Resource) completely randomized block design was used to test the effects of Pacifying Outrage and Occupying Resource on Retaliatory Justice. According to the motivation for Pacifying Outrage, the Damage Level and the Subjective Intent were used to evaluate the Pacifying Outrage; according to the motivation for Occupying Resource, the Moral Disparity and Resource Allocation Constraints were used to evaluate Occupying Resource. The variables are shown in Table 5.

**Table 5:** Experimental Variables of Pacifying Outrage and Occupying Resource.

	**High pacifying outrage**	**Low pacifying outrage**	**Total (*N*)**
High Occupying Resource	*N* = 49	*N* = 71	120
	High Damage Level, High Subjective Intent High Moral Disparity, High Resource Allocation Constraints	Low Damage Level, Low Subjective Intent High Moral Disparity, High Resource Allocation Constraints	
Low Occupying Resource	*N* = 71	*N* = 21	92
	High Damage Level, High Subjective Intent High Moral Disparity, High Resource Allocation Constraints	Low Damage Level, Low Subjective Intent Low Moral Disparity, Low Resource Allocation Constraints	
Total (*N*)	120	92	212

A total of 158 university students from EE University and FF University participated in and completed the experimental study 3, and finally, 152 samples were obtained. Among them, 91 women (59.9%) and 61 men (40.1%); the average age is 20.11 ± 1.17 years old, and the average time is 234.88 ± 98.49 s; there were 64 urban householder (42.1%) and 88 rural householder (57.9%). A 2 (High Deterrence and Control vs. Low Deterrence and Control) × 2 (High Value Confirmation vs. Low Value Confirmation) completely randomized block design was used to examine the effects of Deterrence and Control and Value Confirmation on Retaliatory Justice. According to Deterrence and Control, Reconnaissance Difficulty and Publicity Breadth are used to evaluate Deterrence and Control; according to Value Confirmation, Normative Recognition Level and Communication Effectiveness are used to evaluate Value Confirmation. The variables are shown in Table 6.

**Table 6:** Experimental Variables of Deterrence and Control and Value Confirmation.

	**High deterrence and control**	**Low deterrence and control**	**Total (*N*)**
High value confirmation	*N* = 49	*N* = 71	120
	High Reconnaissance Difficulty, High Publicity Breadth High Normative Recognition Level, High Communication Effectiveness	Low Reconnaissance Difficulty, Low Publicity Breadth High Normative Recognition Level, High Communication Effectiveness	
Low Value Confirmation	*N* = 71	*N* = 21	92
	High Reconnaissance Difficulty, High Publicity Breadth High Normative Recognition level, High Communication Effectiveness	Low Reconnaissance Difficulty, low Publicity Breadth Low Normative Recognition Level, Low Communication Effectiveness	
Total (*N*)	120	92	212

### Experimental results

#### Study 1

Through the independent sample *t*-test, it was found that the Retaliatory Justice score of men (6.91 ± 2.52) was slightly higher than that of women (6.51 ± 1.76), but the difference was not significant (*t* = 1.013, *p* = 0.314). Rural household registration (6.85 ± 2.20) was slightly higher than urban household registration (6.50 ± 2.01), but the difference was not significant (*t* = −0.949, *p* = 0.344). The subjects in the High Pacifying Outrage situation thought that ZHANG's behavior was worse (higher score) and less worthy of forgiveness (lower score), which was significantly different from the subjects in the Low Pacifying Outrage situation. Subjects in the High Deterrence and Control situation believed that ZHANG's behavior would be reported more widely (higher score), which was significantly different from the Low Deterrence and Control situation, but there was no significant difference in the manipulation of Reconnaissance Difficulty. The results of the independent sample *t*-test were also successful, and there were significant differences in the scores of Pacifying Outrage and Deterrence and Control. The statistical results are shown in Table 7.

**Table 7:** Test results of Pacifying Outrage and Deterrence and Control.

**Group**	***N***	**M ±SD**	***t***	***P***
High Damage Level	80	5.53 ± 0.98	7.760	0.000
Low Damage Level	53	3.77 ± 1.44		
High Subjective Intent	80	2.66 ± 1.61	8.417	0.000
Low Subjective Intent	53	4.79 ± 1.29		
High Reconnaissance Difficulty	72	5.44 ± 1.46	−0.251	0.802
Low Reconnaissance Difficulty	61	5.38 ± 1.63		
High Publicity Breadth	72	5.15 ± 1.56	4.232	0.000
Low Publicity Breadth	61	4.05 ± 1.42		
High Pacifying Outrage	80	5.43 ± 1.01	10.447	0.000
Low Pacifying Outrage	53	3.49 ± 1.10		
High Deterrent and Control	72	3.85 ± 1.09	2.636	0.009
Low Deterrent and Control	61	3.34 ± 1.18		

The two-way analysis of variance (ANOVA) was used to test the effects of Pacifying Outrage and Deterrence and Control on Retaliatory Justice. The results showed that only the Pacifying Outrage had a main effect on Retaliatory Justice, and the higher the Pacifying Outrage was, the higher the need for punishment, that is, the stronger the demand for Retaliatory Justice (7.41 ± 1.57, 5.55 ± 2.31, *p* = 0.000). Deterrence and Control did not show a significant effect (6.65±2.05, 6.69±2.18, *p* = 0.733), nor did the interaction effect. The results of ANOVA are shown in Table 8. The results of multiple linear regression analysis showed that only the effect of Pacifying Outrage on Retaliatory Justice showed a main effect, and the interaction between the two was not significant. The statistical results are shown in Table 9. The results show that the higher the level of Retaliatory Justice, the more the subjects seek punishment, and the higher the appeal for Retaliatory Justice.

**Table 8:** Analysis of Variance Results of Pacifying Outrage and Deterrence and Control.

	**MS**	***F***	**df**	***p***	**η^2^**
Constant	5216.493	1428.762	1	0.000	0.917
Pacifying Outrage	107.517	29.448	1	0.000	0.186
Deterrent and Control	0.428	0.117	1	0.733	0.001
Pacifying Outrage × Deterrent and Control	0.771	0.211	1	0.647	0.002
*R^2^*= 0.193, *Adjust R^2^*= 0.174

**Table 9:** Multiple Regression Analysis of the effects of Pacifying Outrage and Deterrence and Control.

	**B**	**β**	***t***	***p***	**Tolerance**	**VIF**
Intercept	2.696	–	3.795	0.000	–	–
Pacifying Outrage	0.913	0.615	8.739	0.000	0.941	1.062
Deterrent and Control	−0.075	−0.041	−0.604	0.547	0.991	1.009
Pacifying Outrage × Deterrent and Control	0.066	0.048	0.686	0.494	0.948	1.055
*R^2^*= 0.399, *Adjust R^2^*= 0.385, *F* = 28.595, *p* = 0.000, *Durbin-Watson* = l.954

#### Study 2

Through the independent sample *t*-test, it was found that the Retaliatory Justice score of men (7.04 ± 1.76) was slightly lower than that of women (7.40 ± 1.83), but the difference was not significant (*t* = −1.427, *p* = 0.155). Rural householder (7.20 ± 1.88) was slightly lower than urban householder (7.31 ± 1.72), but the difference was not significant (*t* = 0.438, *p* = 0.662).

The subjects in the High Pacifying Outrage situation thought that TIAN's behavior was worse (higher score), and the Subjective Intent was less worthy of forgiveness (lower score), which was significantly different from the subjects in the Low Pacifying Outrage situation. The subjects in the High Occupying Resource situation believed that the gap between their own moral status and TIAN should be greater (higher score), and they believed that TIAN should be restricted from having the same rights as others (higher score), which was significantly different from the subjects in the Low Occupying Resource situation. The results of the independent sample *t*-test showed that the operation was successful, and the scores of Pacifying Outrage and Occupying Resource were significantly different, and the results are shown in Table 10.

**Table 10:** Test Results of the Pacifying Outrage and Occupying Resource.

**Group**	***N***	**M ±SD**	***t***	***P***
High Damage Level	92	5.24 ± 0.93	3,673	0.000
Low Damage Level	120	5.29 ± 1.23		
High Subjective Intent	92	2.84 ± 1.81	−2.818	0.005
Low Subjective Intent	120	3.51 ± 165		
High Moral Disparity	120	3.23 ± 1.50	2.936	0.004
Low Moral Disparity	92	2.64 ± 1.35		
High Resource Allocation Constraints	120	4.80 ± 1.52	2.S09	0.005
Low Resource Allocation Constraints	92	4.21 ± 1.54		
High Pacifying Outrage	92	5.50 ± 1.05	4.171	0.000
Low Pacifying Outrage	120	4.89 ± 1.05		
High Occupying Resource	120	4.01 ± 1.13	3.805	0.000
Low Occupying Resource	92	3.42 ± 1.09		

The two-way ANOVA was used to test the effects of Pacifying Outrage and Occupying Resource on Retaliatory Justice. The results showed that only the Pacifying Outrage had a main effect on Retaliatory Justice, and the higher the vindictiveness, the higher the need for punishment, that is, the stronger the demand for Retaliatory Justice (7.78 ± 1.59, 6.84 ± 1.86, *p* = 0.002). Occupying Resource did not show a significant effect (7.44 ± 1.89, 7.00 ± 1.66, *p* = 0.548), nor did the interaction effect. The results of variance analysis are shown in Table 11. There may be an interaction between Pacifying Outrage and Occupying Resource. The results of multiple linear regression analysis show that the Pacifying Outrage significantly affects Retaliatory Justice, and Occupying Resource significantly affects Retaliatory Justice, but the interaction between the two is not significant. The statistical results are shown in Table 12. The results show that the higher the level of Pacifying Outrage, the more the subjects seek punishment, and the higher the appeal for Retaliatory Justice. The higher the Occupying Resource, the more the subjects seek punishment, and the higher the demand for Retaliatory Justice.

**Table 11:** Analysis of Variance Results between Pacifying Outrage and Occupying Resource.

	**MS**	***F***	**df**	***p***	***η^2^***
Constant	8781.642	2854.84	1	0.000	0.932
Pacifying Outrage	30.084	9.780	1	0.002	0.045
Occupying Resource	1.112	0.361	1	0.548	0.002
Pacifying Outrage × Occupying Resource	1.228	0.399	1	0.528	0.002
*R^2^*= 0.070, *Adjust R^2^*= 0.056

**Table 12:** Multiple Regression Analysis of the Effects of Pacifying Outrage and Occupying Resource.

	**B**	**β**	***t***	***p***	**Tolerance**	**VIF**
Intercept	3.343	–	5.763	0.000	–	–
Pacifying Outrage	0.449	0.272	4.011	0.000	0.841	1.189
Deterrent and Control	0.428	0.273	4.073	0.000	0.859	1.164
Pacifying Outrage × Deterrent and Control	−0.033	−0.024	−0.375	0.708	0.943	1.061
*R^2^*= 0.198, *Adjust R^2^*= 0.186, *F* = 17.097, *p* = 0.000, *Durbin-Watson* = 2.172

#### Study 3

Through the independent sample *t*-test, it was found that the Retaliatory Justice score of men (7.72 ± 2.22) was slightly higher than that of women (7.07 ± 2.20), but the difference was not significant (*t* = 1.793, *p* = 0.075). Rural householder (7.59 ± 2.28) was slightly higher than urban householder (6.97 ± 2.11), but the difference was not significant (*t* = −1.713, *p* = 0.089).

The subjects in the High Value Confirmation situation believed that ZHAO showed higher recognition of social value norms and apologized more sincerely to the victim (higher score), which was significantly different from the subjects in the Low Value Confirmation situation. The subjects in the High Deterrence and Control situation thought that ZHAO's behavior was less likely to be discovered (lower score), which was significantly different from the subjects in the Low Deterrence and Control situation, but there was no significant difference in the operation of Propaganda Breadth. The results of the independent sample *t*-test showed that the operation was successful, and there were significant differences in the scores of Value Confirmation and Deterrence and Control. The statistical results are shown in Table 13.

**Table 13:** Test Results of Value Confirmation and Deterrence and Control.

**Group**	***N***	**M ±SD**	***t***	***P***
High Normative Recognition Level	64	5.05 ± 1.64	7.885	0.000
Low Normative Recognition Level	88	2.32 ± 1.75		
High Communication Effectiveness	64	4.59 ± 1.50	9.765	0.000
Low Communication Effectiveness	88	2.38 ± 1.85		
High Reconnaissance Difficulty	84	4.95 ± 1.61	−3.479	0.001
Low Reconnaissance Difficulty	68	5.78 ± 1.33		
High Publicity Breadth	84	4.93 ± 1.51	0.833	0.406
Low Publicity Breadth	68	4.72 ± 1.55		
High Value Confirmation	88	5.65 ± 1.52	10.477	0.000
Low Value Confirmation	64	3.18 ± 1.32		
High Deterrence and Control	84	3.99 ± 0.93	3.428	0.001
Low Deterrence and Control	68	3.47 ± 0.91		

“N” represents the number of people.

The two-way ANOVA was used to test the effects of Value Confirmation and Deterrence and Control on Retaliatory Justice. The results show that only Value Confirmation has a main effect on Retaliatory Justice, and the higher the Value Confirmation, the lower the need for punishment, that is, the weaker the demand for Retaliatory Justice (6.61 ± 2.14, 7.85 ± 2.15, *p* = 0.002). Deterrence and Control did not show a significant effect (7.24 ± 2.16, 7.44 ± 2.31, *p* = 0.831), nor did the interaction effect. The analysis of variance results are shown in Table 14. The Value Confirmation may interact with Deterrence and Control. The results of multiple linear regression analysis show that Value Confirmation significantly affects Retaliatory Justice, and Value Confirmation and Deterrence and Control synergistically affect Retaliatory Justice, but Deterrence and Control has no significant effect on Retaliatory Justice. The statistical results are shown in Table 15. The results show that the higher the Value Confirmation, the lesser the subjects seek punishment, and the lower the appeal for Retaliatory Justice. A simple effect analysis of the interaction effect found that in the context of High Deterrence and Control, the increase in Value Confirmation would reduce the appeal of Retaliatory Justice (*t* = 3.741, *p* = 0.000). However, in the Low Deterrence and Control situation, the increase of Value Confirmation did not significantly reduce the demand for Retaliatory Justice (*t* = 1.153, *p* = 0.253).

**Table 14:** Analysis of Variance Results of Value Confirmation and Deterrence and Control.

	**MS**	***F***	**df**	***p***	***η^2^***
Constant	6719.726	1457.261	1	0.000	0.908
Deterrence and Control	0.210	0.045	1	0.831	0.000
Value Confirmation	45.321	9.829	1	0.002	0.062
Deterrence and Control × Value Confirmation	6.483	1.406	1	0.238	0.009
*R^2^*= 0.037, *Adjust R^2^*= 0.069

**Table 15:** Multiple Regression Analysis of the effect of Value Confirmation and Deterrence and Control.

	**B**	**β**	***t***	***p***	**Tolerance**	**VIF**
Intercept	5.423	–	6.324	0.000	–	–
Pacifying Outrage	0.346	0.293	3.790	0.000	0.992	1.008
Deterrent and Control	0.092	0.040	0.503	0.615	0.956	1.046
Pacifying Outrage × Deterrent and Control	0.230	0.185	2.351	0.020	0.964	1.038
*R^2^*= 0.120, *Adjust R^2^*= 0.103, *F* = 6.755, *p* = 0.000, *Durbin-Watson* = 2.067

## Discussion of empirical results

### Discussion on Pacifying Outrage and Deterrence and Control

The independent sample *t*-test showed that the manipulation of Damage Level, Subjective Intent, and Publicity Breadth were all successful, showing significant differences; but the manipulation of Reconnaissance Difficulty was unsuccessful. This is because university students are not familiar with the situation of “company finance and account review,” and they are not familiar with the behavior of “extracting company cash.” Therefore, the subjects did not respond as expected to the operation of Reconnaissance Difficulty. But overall, the data show a successful operation for Pacifying Outrage and Deterrence and Control.

The results of two-way ANOVA showed that the Pacifying Outrage had a significant effect on Retaliatory Justice. It shows that when the Pacifying Outrage rises, it will lead to more severe punishment (the Damage Level of the crime is greater, and the degree of forgiveness of the crime is lower), showing a stronger demand for Retaliatory Justice. Deterrence and Control cannot significantly affect Retaliatory Justice. So, H1 is validated and H4 is not. The findings suggest that there is some cross-cultural consistency on why people seek punishment. Moral factors are the main reference for the public to measure the severity of punishment (Gonick and Sophie, 2016). The stronger the sense of moral offense, the easier it is to provoke a demand for Retaliatory Justice (Purnell, 2019). However, the jurisprudence logic that “law has the ability to deter potential crimes” in modern law did not respond as expected to the choice of punishment behavior and degree. In today's society, especially in the cyberspace where information spreads rapidly, there are contradictions with the general social cognition (Sawaoka and Monin, 2020; Koak, 2021; Lin and Loi, 2021). The widely accepted view of “what can and cannot be done can only be known by legal sanctions” has given way to the view that “mistakes should be punished.” This may indicate that at the conscious level, individuals are more concerned about the functional value of law, but subconsciously, individuals are more aware of the moral value of law, that is, the legal logic and legal philosophy of deservingness (Lutsenko, 2018; Choi, 2019). It can also be said that for the general public, it may be after the awareness that “the crime deserves it” comes the view that “the law is deterrent.”

The results of multiple linear regression analysis show that there is no linear relationship between Pacifying Outrage and Deterrence and Control, and there is no interaction, indicating that Pacifying Outrage and Deterrence and Control are relatively independent. Although the effect of Deterrence and Control on Retaliatory Justice is not significant, it is worth noting that the impact of the standardized regression coefficient of Deterrence and Control on Retaliatory Justice shows a negative effect, that is, a negative correlation.

### Discussion on Pacifying Outrage and Occupying Resource

The independent sample *t*-tests showed that manipulations for Damage Level, Subjective Intent, Moral Disparity, and Resource Allocation Constraints were all successful, showing significant differences. On the whole, the data also show that the operation for Pacifying Outrage and Occupying Resource was successful.

The results of two-way ANOVA showed that the Pacifying Outrage had a significant effect on Retaliatory Justice. It shows that when the Pacifying Outrage rises, it will lead to more severe punishment (that is, the Damage Level of the crime is greater, and the degree of forgiveness of the crime is lower), showing a stronger demand for Retaliatory Justice. However, Occupying Resource cannot significantly affect Retaliatory Justice. So, H1 is verified again, but H2 is not verified. The research results once again confirm that the Pacifying Outrage has a significant impact on Retaliatory Justice, showing robustness. However, Occupying Resource has not been verified because the moral gap constructed by the “criminal” as a stigma with a strong derogatory connotation is consistent with the individual's perception that their own moral level is better than the average level (Poteat et al., 2013). Therefore, even when the manipulation of Occupying Resource is successful, the individual's appeal to punishment is not affected by the stigma. In other words, even if the Moral Disparity was not constructed by means of stigmatization, and when the subjects chose the severity of punishment, they still showed a strong Retaliatory Justice appeal to the object stigmatized by the offender (Safi et al., 2022). In addition, the current social resource competition is extremely fierce, and individuals will unconsciously participate in social competition (Mor, 2022). The Moral Disparity constructed through criminal stigma has a relatively small effect on the exclusion of competitors, so it cannot respond to individual demands for punishment.

However, the results of two-way ANOVA showed that there may be an interaction between the two motivations. The results of multiple linear regression analysis pointed out that the Pacifying Outrage and the Occupying Resource as the motivation can significantly affect the individual's demands for Retaliatory Justice. Specifically, when the Pacifying Outrage rises, the severity of punishment also increases; when Occupying Resource increases, the severity of punishment also increases, that is, the demand for Retaliatory Justice increases. However, the interaction between the two motivations was not significant. Both Pacifying Outrage and Occupying Resource significantly and positively affect the perception of Retaliatory Justice, and the effects of the two are equal.

### Discussion on Value Confirmation and Deterrence and Control

The independent sample *t*-test showed that the operation of Reconnaissance Difficulty, Norm Recognition Level, and Communication Effectiveness was relatively successful, showing significant differences. However, the operation of Publicity Breadth was not successful, because the scene of the traffic accident was not unfamiliar to the subjects. The operation of the Publicity Breadth in the situational materials may have a great overlap with the publicity and education of traffic accidents that the subjects noticed on a daily basis. To some extent, the subjects were “desensitized” to the media communication of traffic accidents. Therefore, the subjects did not respond as expected to the manipulation of Publicity Breadth. But overall, the data show that the Deterrence and Control and Value Confirmation operation were successful.

The two-way ANOVA results show that Value Confirmation has a significant effect on Retaliatory Justice. It shows that when the Value Confirmation decreases, it will lead to more punishment (the perpetrator still does not recognize and disagree with social value norms), showing a higher pursuit of Retaliatory Justice, while Deterrence and Control cannot significantly affect Retaliatory Justice. So, H3 is validated and H4 is not. Deterrence and Control has no significant effect on Retaliatory Justice, showing robustness. Value Confirmation represents the re-identification and re-confirmation of the social value norms recognized by the mainstream of the society, and it is a kind of behavior constraint expectation based on value. At the same time, Value Confirmation also represents a positive and forward judicial form, that is, Restorative Justice, which is a new turn in modern legal thinking and judicial practice that is different from Deterrence and Control. However, the intuitive online public opinion shows that the public has less recognition of the Restorative Justice and even has a sense of resistance (Jeffries et al., 2021). This is related to the view that “judicial trials should also have an explanation to the public,” that is, judicial trials also have social effects. But empirical data show social support for Restorative Justice. This kind of support can be seen as a psychological expectation for the judicial system and judicial trials to exert positive social benefits, and it can also be seen as a good hope in the hearts of the people for justice to reshape social morality and social value (Gavrielides, 2022).

However, the results of two-way analysis of variance clearly showed that there may be an interaction between the two motivations. The results of the multiple linear regression analysis also proved that only Value Confirmation has a main effect; but unlike the results of the analysis of variance, the interaction of the two motivations was also significant. Specifically, the main effect represents that when the Value Confirmation increases, the severity of punishment will decrease; the interaction effect represents that the Value Confirmation and Deterrence and Control synergistically affect the level of the subjects' demands for Retaliatory Justice. Through the simple effect test, it is found that only in the High Deterrence and Control situation, when the Value Confirmation increases, the severity of punishment will decrease, that is, the appeal for Retaliatory Justice will decrease. However, in the Low Deterrence and Control situation, the increase or decrease of Value Confirmation does not significantly affect the demands for Retaliatory Justice (Mills et al., 2019; Decker et al., 2022). This difference may be related to the nature of Restorative Justice's target intervention object. As a judicial practice, Restorative Justice is often aimed at juvenile offenders and light offenders, which means that the punishment for offenders is mainly re-education. Only in the High Deterrence and Control situation, Restorative Justice's judicial practice can exert the greatest social benefit. On the one hand, it can achieve specific deterrence for offenders, and on the other hand, it can give the public an explanation. In the context of Low Deterrence and Control, Restorative Justice's judicial practice often only concerns the perpetrator and the victim and does not produce more widely known social benefits (Tapp et al., 2020).

## Rheological path of the psychological motivation of Retaliatory Justice

**Figure 3:**
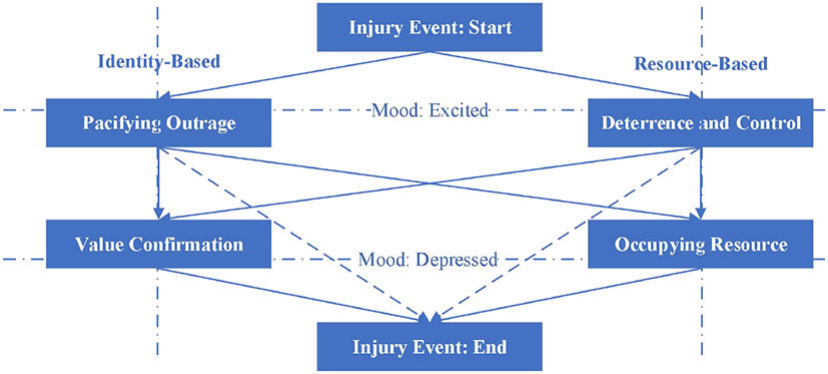
Rheological paths of the psychological motivations of Retaliatory Justice.

As we know from the above, the psychological motivation of Retaliatory Justice is rheological, and the main source is the directionality, fluidity, and fluctuation of the emotions it contains. The types of the psychological motivation of Retaliatory Justice are constructed around the dimensions of “Identity vs. Resource” and “Self vs. Society.” Therefore, with the direction and flow of emotions as clues, with resource and identity as reference, and from the perspective of self and society, we will explore the possible rheological paths of the psychological motivation of Retaliatory Justice, and the social effects produced by the rheology of motivation. According to the ups and downs of emotions, the Pacifying Outrage and Deterrence and Control are premised on the occurrence of injury events, which is an activated state, showing an upward trend. Occupying Resource and Value Confirmation are based on the end of the injury event, which is a state of gradual recovery, showing a downward trend. Different psychological motivations of Retaliatory Justice show the alternation, superposition, and recurrence, and their emotions are also similar. However, the development and evolution model of injury events is specific, that is, only the beginning and ending states are analyzed, and the complexity and rheology of the event development process are omitted. Therefore, Retaliatory Justice has a total of five rheological paths of psychological motivation, which are named as Social Integration Path, Social Differentiation Path, Social Harm Path, Social Trust Path, and Social Depression Path. Figure 3 shows the rheological paths of the psychological motivation of Retaliatory Justice.

### Social Integration Path: From Pacifying Outrage to Value Confirmation

Social Integration Path begins with the Pacifying Outrage and ends with the Value Confirmation. It is a path that refers to moral norms, value norms, and social norms. The emotional core of Pacifying Outrage is the feeling of indignation, which is generated by the occurrence of harmful behaviors. The moral value norms generally recognized by the social collective are broken, and the moral feelings of individuals are offended, so the emotions are constantly gathering, intense, and rising. The emotional core of Value Confirmation is the sense of value. Due to multi-party communication, consensus and other ways of recognizing value, the indignation is reduced and transformed into a sense of value, recognizing, accepting, and agreeing with the meaning and value of social norms, thus calming the intense emotional experience. A complete Social Integration Path (a complete process of emotional change) represents that the perpetrators, victims, and the public are healed, not only the harmful feelings are understood, but also the reshaping of values, and the guidance and reshaping of social behaviors. Therefore, the Social Integration Path is a positive and healthy development path of psychological motivation of Retaliatory Justice.

Social Integration Path is also a path of “from Self to Society,” because the generation of Pacifying Outrage often stems from the challenge of individual moral cognition, which is the emotional state of the individual level. However, due to the increasing attention and discussion of the injury incident, the individual's indignation has continuously converged into a social emotional force. Each emotional subject is attached to each other, amplifying the social impact of the injury event, and the individuality of the emotional subject is dissolved in the atmosphere of social emotions. This response is not directed at the individual perpetrator, but at all members of society. Through the reshaping of the perpetrator's concept and behavior, the re-strengthening and re-affirmation of the social value and social moral value of all members of the society are realized. When the Value Confirmation based on restorative logic is satisfied, the relationship between the perpetrator and other social members can be repaired, and the common social value norm is the “glue” of the relationship repair. Judicial practice centered on value identification, concept identification, and moral identification guides and promotes value norm identification from Self to Society, from individual to collective, and from perpetrators to society as a whole. The emotional evolution process from indignation to sense of value represents a Social Integration Path with the rheology of psychological motivation.

### Social Differentiation Path: From Deterrence and Control to Occupying Resource

Social Differentiation Path begins with Deterrence and Control and ends with Occupying Resource. It is an evolutionary process of psychological motivations with reference to resources, power, status, and interests. The emotional core of Deterrence and Control is anxiety. When an injury occurs, it represents a kind of anxiety and worry about potential injury, and it is a process of emotional experience from scratch. The emotional core of Occupying Resource is self-protection. The anxiety that was aroused before was dissolved in the moral superiority, and the emotional experience showed a gradual calming development trend. A complete Social Differentiation Path (a complete process of emotional change) can be seen as increasing the negative experience of society as a whole. On the one hand, bystanders treat criminals with hatred, hostility, and precaution, and their psychological experience is dominated by anxiety, worry, and fear. On the other hand, former criminals suffer from stigma and secondary negative experiences caused by stigma, such as social exclusion and so on. Therefore, the Social Differentiation Path is a negative rheological path, which distinguishes the social members as a whole into different subgroups.

Social Differentiation Path is also a path of “from Society to Self.” First of all, the social deterrent effect of calling for legal sanctions is rational from a global perspective, representing the interests of safeguarding the overall society, and is the social aspect of law. This is different from the individual side of the law (mediating specific disputes, making specific decisions, affecting individual lives). However, Social Differentiation Path focuses on “self-protection.” It is true that the behavior of protecting oneself from harm is reasonable and justified, but it should be within a reasonable limit and should not be premised on harming others. In other words, self-protection through stigma and marginalization is not justified. Therefore, the foothold of social differentiation still seems to have an egoistic gray line, which is not politically correct. On the other hand, does Social Differentiation Path represent an immoral side? Maybe not. Although Social Differentiation Path is realized through the Stigma Theory, the social problems it reflects are worth pondering. For example, are the penalties too light for a particular crime? Are there any omissions in the law? As far as society is concerned, although Social Differentiation Path is not so “healthy,” it actually reflects the places that institutional design has not yet taken care of. Generally speaking, Social Differentiation Path is based on self-interest, which weakens the integrity of the social collective and brings challenges to the positive and orderly development of society.

### Social Harm Path: From Pacifying Outrage to Occupying Resource

Social Harm Path begins with Pacifying Outrage and ends with Occupying Resource, taking individual feelings as a reference and as a guide for action. The emotional core of Pacifying Outrage is the indignation, which is the emotional activation of injury events and behaviors, and the subjective psychological feeling and emotional experience of being injured and suffering morally. Emotions are gradually deepened in the short period following the event. The emotional core of Occupying Resource is superiority, with stigma as the logic, and possession of resources, status, and power as the purpose. When the recognition and approval of a higher moral status is obtained, the psychological experience is in a state of contentment and stability, which is the subjective state at the end of the event. Furthermore, among the two groups constructed by the difference between stigma and moral status, those with high status have reasons to rationalize the harm and finally reach the “happy” ending that the perpetrator is harmed. Therefore, Social Harm Path is based on the logic of revenge in response to injury, and it is a process from the induction of individual emotions to the recovery of individual emotions.

Social Harm Path is still a path of “from Self to Self.” This is because the path is always wandering at the individual level, always taking the individual's emotional feelings as clues, and always taking the individual as the basic starting point. Social Harm Path originates from the individual's moral perception being offended, which leads to anger; and the individual imposes “sanctions” on the subject of the harm in the name of morality and justice and thus is appeased. Social actions under Social Harm Path will increase the overall injury volume of the society. A society that is already negatively affected by harmful behavior will hurt more people through further social exclusion and social stigma, which is one of the reasons why this path is called Social Harm Path. More importantly, Social Harm Path represents a route from personal anger to personal retaliatory action. Social Harm Path often occurs when institutional punishment or institutional control fails. That is to say, official, statutory, and institutional punishment cannot satisfy the people's social and psychological needs, nor can they calm the people's indignation at punishing the perpetrators. Therefore, at this time, the people are more likely to take up the responsibility of punishing criminals in the name of justice. This progressive process from indignation to revenge is similar to revenge in primitive society, or self-help relief. However, it should be pointed out that Social Harm Path in this study is to discuss the rationality and legitimacy of harm as a bystander. Obviously, the bystander does not have the complete moral reason to give the perpetrator a heavy counterattack like the victim, let alone the complete legitimacy of hurting others. Therefore, for bystanders, only a roundabout, tactful, and gentle way can be used to “harm.” This kind of harm can be slander, slander, or rumors, with the ultimate goal of further deepening the infamous character of the perpetrator. This is as comforting as the stagnation in the individual's heart is finally relieved, bringing him a sense of satisfaction, justice, and superiority. It can be seen that the entire social harm path begins with harm and ends with harm, and mutual harm is the essential feature of this path.

### Social Trust Path: From Deterrence and Control to Value Confirmation

Contrary to Social Harm Path, Social Trust Path is a macropath, from Deterrence and Control to Value Confirmation. The emotional core of Deterrence and Control is anxiety, which is diffuse, shallow, and weakly arousing anxiety about the potential harm to oneself. Deterrence and Control reflects the individual's need for the social deterrent effect of punishment, from the perspective of the social function of punishment. The emotional core of Value Confirmation is the sense of value, which is the individual's recognition of social norms, social values, and social morals. Practicing individual values in a practical way maintains the unity of knowledge and action. The two motives represent the process of cognition from inconsistency to consistency, that is, from a state of dissonance to a state of calm. Therefore, it can be considered that the complete Social Trust Path (the complete emotional change process) is a logical path framed by law. It not only recognizes the deterrent effect of the law, but also recognizes the educational function brought about by judicial practice.

Social Trust Path is also a “from Society to Society” path. The legal deterrence function represented by Deterrence and Control and the legal education function represented by Value Confirmation are unified in the Social Trust Path. Although the deterrence function of the law and the educational function are two different functions, the two functions are combined into one, which is consistent with the public's social and psychological cognition. That is, the two functions are in a progressive relationship from here to there. Moreover, the deterrence function of the law and the educational function of the law are both based on society and can be recognized by the public. In other words, the public wants to see that legal sanctions can deter “bad guys” and educate them. This kind of social psychology is universal, and most members of society will not oppose the legal system, nor will they oppose the deterrent and educational functions of the legal system. Thus, the individual needs for Deterrence and Control and Value Confirmation are unified at the social level, and individuality dissolves in it. Therefore, the Social Trust Path is a macro-, social, and ideal paths.

### Social Depression Path: From Pacifying Outrage, Deterrence and Control to emotional disappearance

Social Depression Path is a different form of existence than the previous four paths. Its particularity lies in that it has only a starting point, but no identifiable endpoint, that is, the psychological motivation of Retaliatory Justice “disappears” in the rheology. Social Depression Path begins with the occurrence of a traumatic event, that is, when social emotions are aroused. But there is no clear end to Social Depression Path. Whenever an injury event occurs, it is often difficult for bystanders to have a continuous substitution for the event, and their attention to the injury behavior, object, and result can only be maintained for a period of time. But over time, the bystander's attention is distracted by the rest of daily life, and the cognitive resources and emotional energy are no longer devoted to an unrelated event. Therefore, the unsatisfied social psychological demands are suppressed into the subconscious, and the perception of bystanders will not be activated again until the next event is mentioned or a similar event occurs. In addition, Social Depression Path may also be due to the fact that bystanders clearly know that their abilities cannot lead to changes in the development of events, and their actions have no influence on the injury event. Therefore, under the strong emotional impact, bystanders are likely to choose rationalized cognitive strategies to suppress unsatisfied social and psychological demands. Hence, the transfer of cognitive resources and the inability to do anything about the occurrence of injuries may lead to the occurrence of Social Depression Path, and Social Depression Path often occurs unconsciously in the information explosion network society.

Social Depression Path takes “nothing to do” as the end of the rheology of psychological motivation. But in fact, the negative impact of Social Depression Path on social development may only emerge after a long period of time, just like the butterfly effect. According to the previous interpretation of the rheology of the psychological motivation, the deduction of the psychological motivation of Retaliatory Justice develops along emotional cues. However, Social Depression Path is an emotional state of “up and down.” This is also known as repression, the repression of emotional and psychological needs in the subconscious. In essence, the rheology of the psychological motivation of Retaliatory Justice is a process in which social emotions find an outlet. However, in the rheology of Social Depression Path, social emotions are not effectively relieved, but are limited to conscious perception. It can be said that the negative social emotions caused by the injury event have been suppressed, and its power is accumulating “quietly.” Once it encounters the next exit, it may erupt in a concentrated manner, which can also be understood as a rebound in social emotions. This rebound process, if there is no buffer, can easily develop into a mass incident. Once individuals with high emotions gather into groups, the group is prone to irrationality. When the social emotions caused by injury events are not “digested,” in the vicious circle of accumulation, repression, re-accumulation, and re-repression, one day it will develop into a mass incident, increasing the cost of social governance. The number of times Social Depression Path is repeated represents the number of times the public perceives Retaliatory Justice. When the number of times is more, it shows that the injustice of retaliation is strengthened more. Therefore, in extreme cases, Social Depression Path is likely to destroy or even subvert the stable state of individual moral emotions, cognition, and concepts. Because, the pursuit of fairness and justice is the basic psychological need of human beings, and punishment for making mistakes is the basic principle of morality since ancient times. If Retaliatory Justice is repeatedly intensified, it will naturally make the road of building the rule of law less effective and bumpy.

## Conclusion

1. From the two dimensions of “Self vs. Society” and “Identity vs. Resource,” a four-dimensional motivation model of Retaliatory Justice is constructed, namely, Pacifying Outrage, Value Confirmation, Occupying Resource, and Deterrence and Control.

2. Through the situational experiment, the justice four-dimensional motivation model of Retaliatory Justice was empirically verified. The results show that the two motivations based on identity are significant, namely, Pacifying Outrage and Value Confirmation are significant; the two motivations based on resource are partially significant, namely, Occupying Resource and Deterrence and Control are partially significant.

3. Coupling the development of events, emotional changes, and the psychological motivation of Retaliatory Justice, five rheological paths of the psychological motivation of Retaliatory Justice are sorted out, namely, Social Integration Path, Social Differentiation Path, Social Harm Path, Social Trust Path, and Social Depression Path.

4. The four-dimensional model and rheological paths of the psychological motivation of Retaliatory Justice are discussed to guide the improvement of social morality, the cultivation of judicial trust, and the construction of psychological service system.

### Shortcomings

This study has explained the motivational structure and theoretical connotation of Retaliatory Justice, but there are still several shortcomings as follows.

1) Sociology, Psychology, Law, and Communication must give different answers to the question of what factors influence the psychosocial motivation of Retaliatory Justice, so there is still a need for multidisciplinary thinking under the interdisciplinary topic of Retaliatory Justice.
2) In the follow-up study, this study needs to continue to test the Four-Dimensional Psychological Motivation Model and make theoretical corrections and should also strengthen the control of irrelevant variables and carry out the evaluation of the effectiveness of the Four-Dimensional Psychological Motivation of Retaliatory Justice.
3) This study concludes with an attempt to find the possibility of theoretical conceptions on real system construction, but it is by no means a simple process for social moral construction, judicial trust cultivation, or psychological service system construction. We also need to go deeper into social practice, enter into the operation mechanism of media and judiciary, and find the path of psychological and sociological governance.

## Data availability statement

The raw data supporting the conclusions of this article will be made available by the authors, without undue reservation.

## Author contributions

XL: writing—review and editing, data curation, and formal analysis. XZ: investigation, formal analysis, and supervision. BW: resources, visualization, and supervision. All authors contributed to the article and approved the submitted version.
